# The contingent use of cell-free fetal DNA for prenatal screening of trisomies 21, 18, 13 in pregnant women within a national health service: A budget impact analysis

**DOI:** 10.1371/journal.pone.0218166

**Published:** 2019-06-12

**Authors:** Federico Prefumo, Davide Paolini, Giulia Speranza, Marilena Palmisano, Matteo Dionisi, Lamberto Camurri

**Affiliations:** 1 ASST degli Spedali Civili di Brescia, Brescia, Italy; 2 Medical & Market Access Department, Roche Diagnostics, Monza, Italy; 3 Medi Saluser, Parma, Italy; University of Hong Kong, CHINA

## Abstract

**Objective:**

Non-invasive prenatal testing (NIPT) based on cell-free fetal DNA (cffDNA) is highly accurate in the detection of common fetal autosomal trisomies. Aim of this project was to investigate short-term costs and clinical outcomes of the contingent use of cffDNA for prenatal screening of trisomies 21, 18, 13 within a national health service (NHS).

**Methods:**

An economic analysis was developed from the perspective of the Italian NHS to compare two possible scenarios for managing pregnant women: women managed according to the Standard of Care screening (SoC) vs a cffDNA scenario, where Harmony Prenatal Test was introduced as a second line screening choice for women with an “at risk” result from SoC screening.

**Results:**

The introduction of cffDNA as a second line screening test, conditional to a risk ≥ 1:1,000 from SoC screening, showed a 3% increase in the detection of trisomies, with a 71% decrease in the number of invasive tests performed. Total short-term costs (pregnancy management until childbirth) decreased by € 19 million (from € 84.5 to 65.5 million).

**Conclusion:**

The adoption of the Harmony Prenatal Test in women resulting at risk from SoC screening, implied a greater number of trisomies detection, together with a reduction of the healthcare costs.

## 1. Introduction

Prenatal diagnostic techniques, including instrumental and laboratory investigations, have the specific aim to monitor the development of the embryo.

Chorionic villus sampling (CVS) and amniocentesis are commonly performed as invasive procedures for prenatal diagnosis [1], aimed at identifying prenatal chromosome abnormalities, such as trisomy21, trisomy18 and trisomy13. These procedures require informed decision-making with regard to pregnancy management. During the last 30 years, non-invasive prenatal screening has been developed in order to introduce non-invasive genetic techniques. These prenatal tests are based on the analysis of biochemical markers in the maternal blood, in combination with ultrasound examinations [2]. The cell free fetal DNA (cffDNA)-based non-invasive prenatal test (NIPT) shows high accuracy in the detection of common fetal autosomal trisomies, especially trisomy-21. NIPT is performed on a blood sample of the pregnant woman. It has been demonstrated that blood contains a certain amount of cell free DNA (cfDNA) deriving from cell lysis. Additionally, cell free fetal DNA (cffDNA) is present in pregnant women’s blood beginning with the fifth week of gestation; cffDNA derives from the lysis of placental cells and clears from the maternal system within hours of giving birth. NIPT is not a diagnostic test, but a screening test, based on direct DNA analysis. The use of dedicated algorithms allows to define the probability of fetal trisomies or aneuploidies of sex chromosomes, selectively analyzing the number of the cffDNA fragments [2].

Harmony Prenatal Test is a cffDNA-based NIPT that can be carried out after 10 weeks of pregnancy and it is characterized by an accuracy of 99.95% for trisomy 21 [3]. The purpose of the Harmony Prenatal Test is to provide correct information to expecting parents, because subsequent choices and decisions are based on accurate knowledge and on protocols that do not endanger the pregnancy. NIPT tests have rapidly captured an increasing market share, giving rise to substantial reductions in the number of CVS and amniocentesis, and consequently in the number of rare complications and miscarriages [4].

In Italy, NIPT tests, like the Harmony Prenatal Test, are not currently reimbursed by the National Health Service (NHS). The aim of this study was to evaluate the economic impact from the Italian NHS payer’s perspective of the use of the Harmony Prenatal Test. The study assessed the impact of the second line introduction of NIPT for pregnant women, who performed Standard of Care screening (SoC), compared to the current clinical practice. In particular, this study evaluates the clinical value and cost consequences of using Harmony Prenatal Test for the detection of trisomies 21, 13 and 18.

## 2. Methods

A health economics model, with one-year time horizon, was developed from the perspective of the Italian NHS, customizing patient pathways and management, and cost inputs according to Italian setting. In this model, pregnant women were followed during the screening process and up to the delivery, taking into account the screening tests, invasive diagnostic procedures, and complication management procedures.

A form report addressed to Italian Experts was developed in order to set up and identify the epidemiology and the target population, clinical outcomes and the diagnostic processes with the relative costs for prenatal screening of trisomies 21, 18, 13.

**Fig 1:**
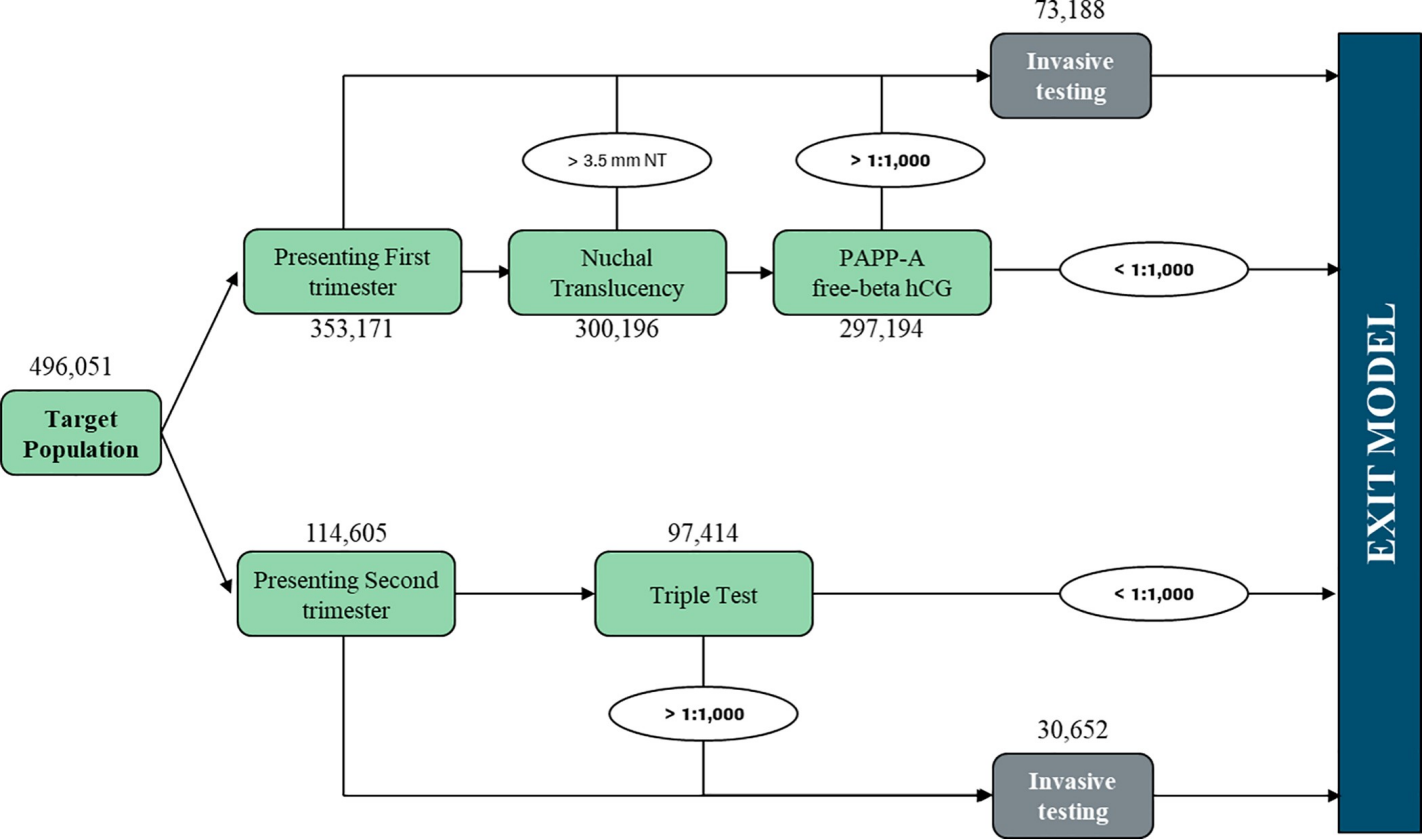
“SoC scenario” patient pathway.

**Fig 2:**
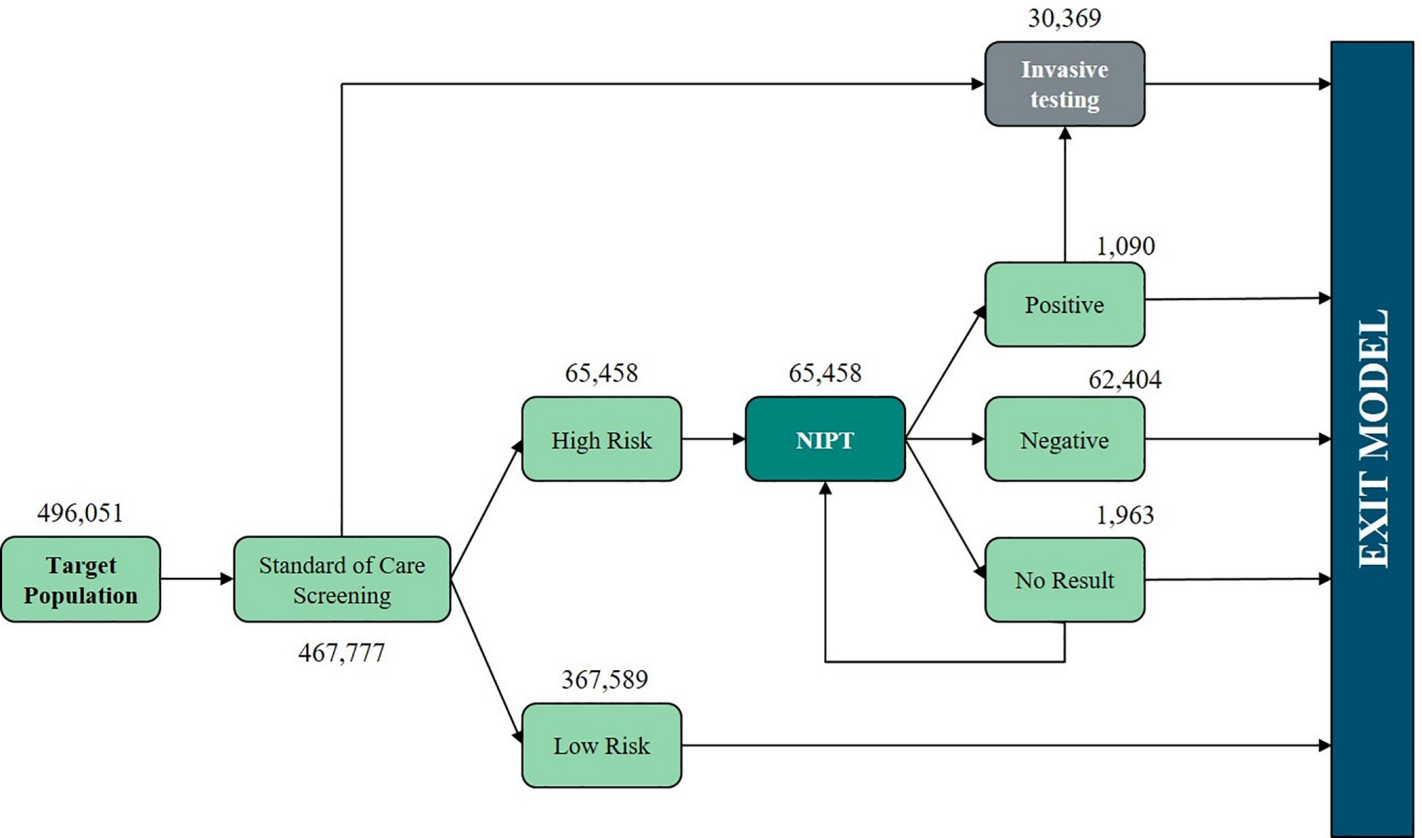
"cffDNA scenario" patient pathway.

The economic evaluation was implemented according to the comparison of two different patient pathways:

- SoC scenario: the current clinical practice based on the standard of care screening (Fig 1);
- cffDNA scenario: the introduction of the Harmony Prenatal Testing in the current clinical practice (Fig 2) as a second line test conditional to a risk ≥ 1:1,000 from SoC screening.

The SoC scenario is composed of (i) first trimester screening (FTS), which includes nuchal translucency ultrasound (NT) and maternal serum testing (PAPP-A and free beta-hCG), (ii) second trimester screening (STS), composed of the triple test (i.e. alpha-fetoprotein, estriol and hCG), and (iii) integrated test (i.e. FTS and STS combined screening). The cffDNA scenario provides the introduction of cffDNA testing as a second line test (also termed contingent screening) conditional to a risk ≥ 1:1,000 from SoC, and it represents an innovative pathway for the management of a woman's pregnancy.

Dedicated form reports were used to collect data and to define the assumptions necessary for the adaptation of the core model of the economic analysis; these reports were administered to a gynecologist and a cytogeneticist and they were structured in five different sections, in order to define the following items:

1. Target population and epidemiology;
2. Patient management and clinical pathway;
3. Test performance data;
4. Procedures and related costs;
5. Management of complications.

Net impact was calculated as the difference between direct healthcare costs associated with the diagnosis and management of pregnant women in the cffDNA scenario and in the SoC scenario. In this budget impact analysis, the following clinical and economic outcomes were calculated for both analyzed scenarios: number of pregnant women tested, detection rates of trisomies (according to the relative level of sensitivity and specificity, Table 1), number of invasive tests per diagnosed aneuploidy, number of unnecessary invasive tests due to false positives, screening costs (i.e. NT, FTS, STS, and NIPT),invasive testing costs (i.e. CVS and amniocentesis), costs for ancillary care (i.e. procedure-related miscarriage, procedure-related complications, termination of pregnancy, genetic counseling, and additional physician visits for at risk screens). Total testing costs and total short term costs were estimated: total testing costs related to the sum of screening costs and invasive testing costs (see Table 2); total short term costs consisted in the sum of testing costs plus ancillary care costs.

**Table 1:** SoC and harmony prenatal test parameters.

		T21	T18	T13
**FTS [5]**	***Sensitivity (95% CI)***	**88.00** (50.00–100.00)	**90.00** (33.00–100.00)	**78.00** (57.00–100.00)
	***Specificity (95% CI)***	**95.00** (91.00–99.00)	**94.00** (94.00–100.00)	**92.00** (92.00–96.00)
**STS [5]**	***Sensitivity (95% CI)***	**80.50** (60.00–100.00)	**63.50** (53.00–86.00)	**50.00** (1.00–99.00)
	***Specificity (95% CI)***	**90.50** (46.00–96.00)	**99.25** (99.64–100.00)	**64.00** (61.00–67.00)
**Harmony Prenatal Test [6]**	***Sensitivity (95% CI)***	**99.30** (97.90–99.80)	**97.40** (93.40–99.00)	**93.80** (79.90–98.30)
	***Specificity (95% CI)***	**99.96** (99.92–99.98)	**99.98** (99.95–99.99)	**99.98** (99.94–99.99)

**Table 2:** Costs considered in the budget impact analysis [7,8].

***Screening Costs***		
NT ultrasound	€	30.99
First trimester serum testing	€	42.46
Second trimester screening	€	23.33
Harmony Prenatal Test	€	300.00
***Diagnosis Costs***	*** ***	
Chorionic villus sampling (CVS)	€	579.89
Amniocentesis	€	360.05
***Ancillary Care Costs***		
Treatment for rare complications	€	2,452.00
Treatment for leakage	€	1,376.00
Treatment for procedure-related miscarriage	€	1,061.00
Genetic counseling	€	20.66
Additional physician visit	€	12.91

### 2.1 Inputs variables and modeling assumptions

A systematic literature review and expert opinion validation were conducted to collect data input and to describe the clinical pathway based on the Italian real clinical practice. The national Italian Essential Levels of Care (*Livelli Essenziali di Assistenza [9]*) no longer recognize advanced maternal age (≥ 35 years) as an indication for invasive prenatal diagnosis. According to National antenatal care guidelines [10], FTS has to be offered to all women interested in trisomy screening. If a woman has her first antenatal care appointment too late to organize FTS, STS is then offered. Invasive prenatal diagnosis is offered in pregnancies at risk of Mendelian conditions, and in women with a prior history of trisomies 21, 18, or 13, at an estimated prevalence of 1.01% [11].

**Fig 3:**
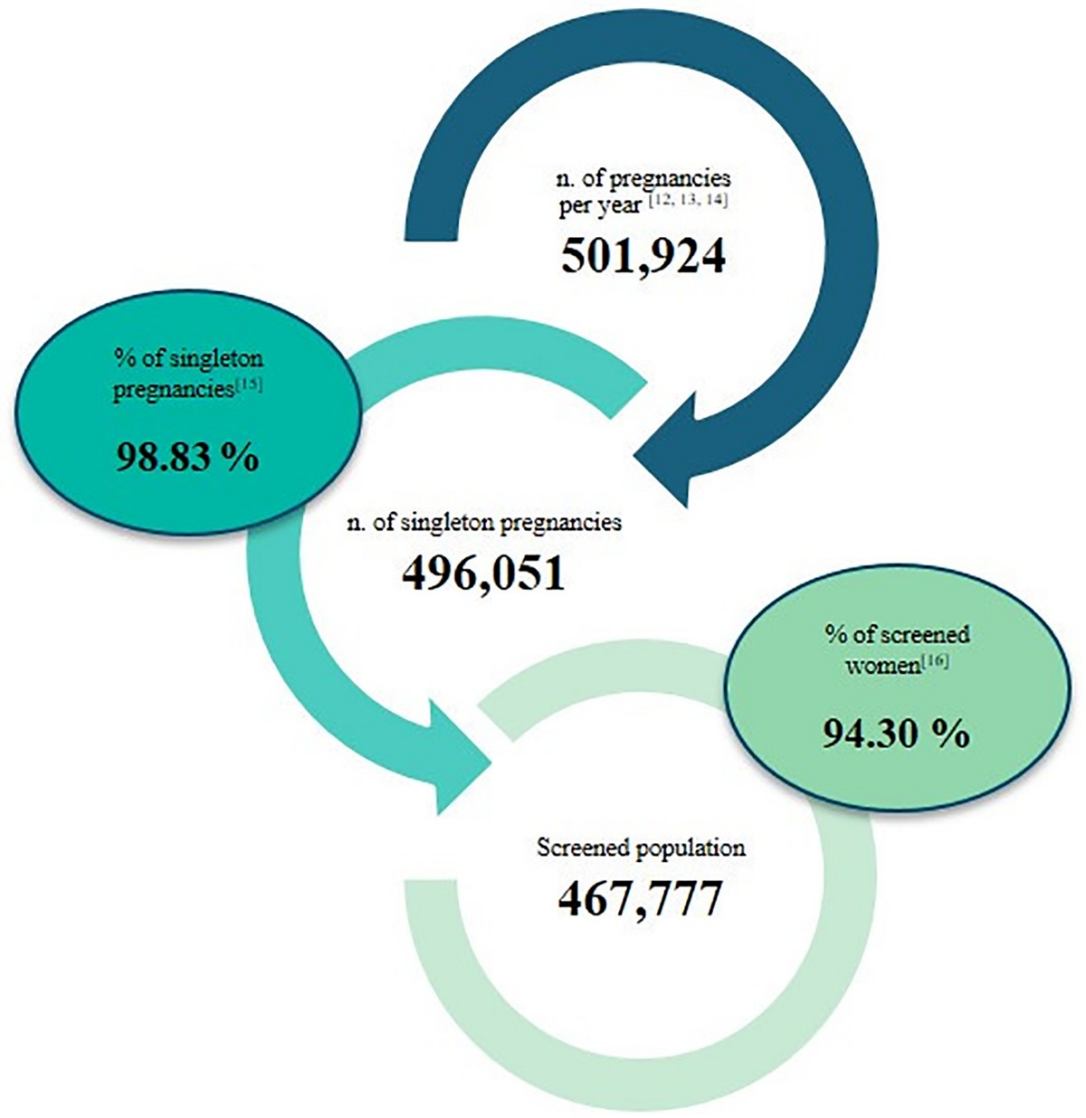
Population flow.

In both scenarios, the health economics model estimates a total number of 501,924 pregnancies reaching 10-week gestation each year [12,13,14], with a singleton pregnancies percentage of 98.83% [15]. The SoC scenario assumes that 94.30% of singleton pregnant women (467,777) undergo prenatal screening each year [16] (Fig 3). According to screened pregnant women, 75% of them undergo FTS with a unit cost of € 42.46, 24.5% participates in STS with a relative cost of € 23.33, and a smaller portion (0.5%) participates in both FTS and STS (integrated test) [7,17].

According to the SoC scenario, 15% of women presenting for FTS goes directly to invasive testing before NT ultrasound. Conversely, in cffDNA scenario it was assumed that no women presenting for FTS go directly to invasive diagnosis, but 100% undergo NT ultrasound at a cost of €30.99 [7,17]. According to both scenarios, 15% of pregnant women presenting in the second trimester directly undergo invasive testing and the others undergo STS. All these procedures are performed in an outpatient setting and, according to NHS reimbursement, specific relative outpatient tariffs were identified.

Invasive diagnostic tests are recommended after a high-risk result from current screening strategies (risk ≥ 1:300 at FTS or ≥ 1:250 at STS [9]) in order to confirm analysis results. In the SoC scenario 87.50% of women choose to perform an invasive test after a risk result from FTS and STS, considering a cost of € 579.89 for CVS and € 360.05 for amniocentesis [7]. Ancillary care costs are related to risks of abortion and complications related to invasive testing: 0.5% could have a miscarriage at a cost of € 1,061 [8,18]; another 1% undergo additional care related to the diagnosis/suspicion of amniotic fluid leakage at a cost of € 1,376 [8,19] and, another 1% receives care related to the management of additional rare complications with a cost of € 2,452[8]. The percentage of women with an “at risk” results receiving genetic counseling prior to invasive testing is 30%, whether they come from FTS or STS. An additional specialist visit is considered at a cost of € 12.91 per each patient with increased risk results [7].

In the cffDNA scenario, the Harmony Prenatal Test was introduced as a second line test for women with a risk of developing trisomies ≥ 1:1,000 after SoC screening. Of these, 10.52% of pregnant women with a very high risk (e.g. 1:10) to develop trisomies from FTS and STS in SoC screening, directly underwent invasive testing [20,21].

82.69% of women with a risk ≥ 1:1,000 underwent cffDNA testing with an estimated cost of € 300 per test [20,22]. The cost, related to the re-test that concerns 3% of women undergoing cffDNA because of no result, has been also considered. In case of double “no result” from cffDNA testing, the screening continues with the SoC approach. It was assumed that 90% of women with an “at risk” result from cffDNA would opt for invasive testing. The main inputs and assumptions related to the patient flow are reported in Table 3.

**Table 3:** Patient flow inputs and assumptions [17, 18, 19, 20, 21,22].

***SoC and cffDNA scenario***	
75.00%	Pregnant women undergoing FTS
24.50%	Pregnant women undergoing STS
0.50%	Pregnant women undergoing both FTS and STS
0.50%	Procedure-related miscarriage
1.00%	Procedure-related leakage of amniotic fluid
1.00%	Procedure-related rare complications
***SoC scenario***	
15.00%	Pregnant women undergoing directly invasive procedure before NT or STS
87.50%	Pregnant women undergoing invasive testing after a risk result from FTS or STS
***cffDNA scenario***	
100.00%	Pregnant women undergoing NT
15.00%	Pregnant women undergoing directly invasive test before STS
10.52%	Pregnant women undergoing invasive testing after a risk result from FTS or STS
82.69%	Pregnant women undergoing cffDNA testing after a risk result from FTS or STS
90.00%	Pregnant women undergoing invasive testing after a risk result from cffDNA

## 3. Results

**Fig 4:**
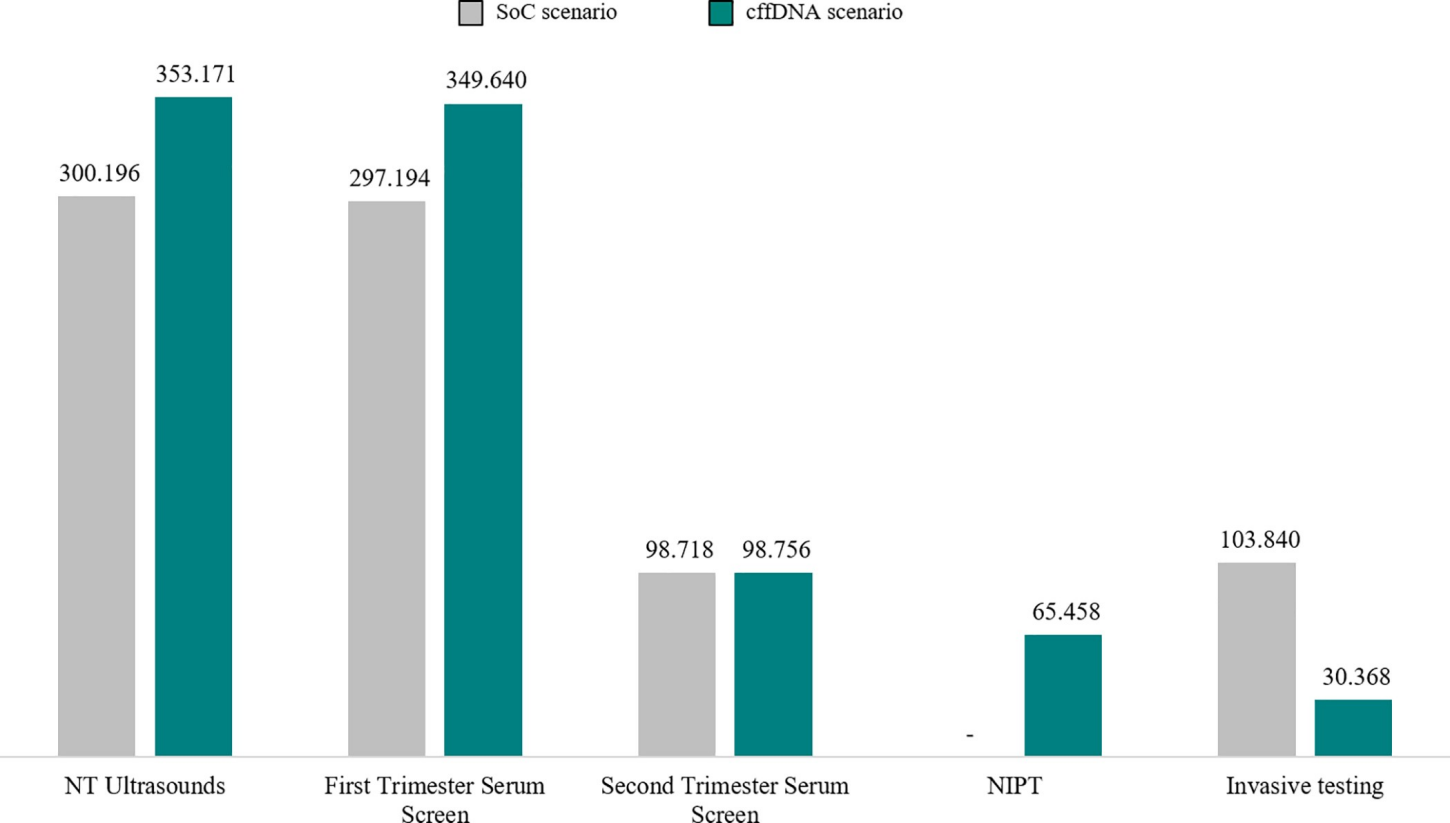
Number of pregnant women tested.

The introduction of cffDNA testing as a second line screening produces an increase in the number of screening procedures (from 696,107 in SoC scenario to 801,567 in cffDNA scenario). In detail, 353,171 NT ultrasound, 349,640 FTS, and 98,756 STS (Fig 4).

**Fig 5:**
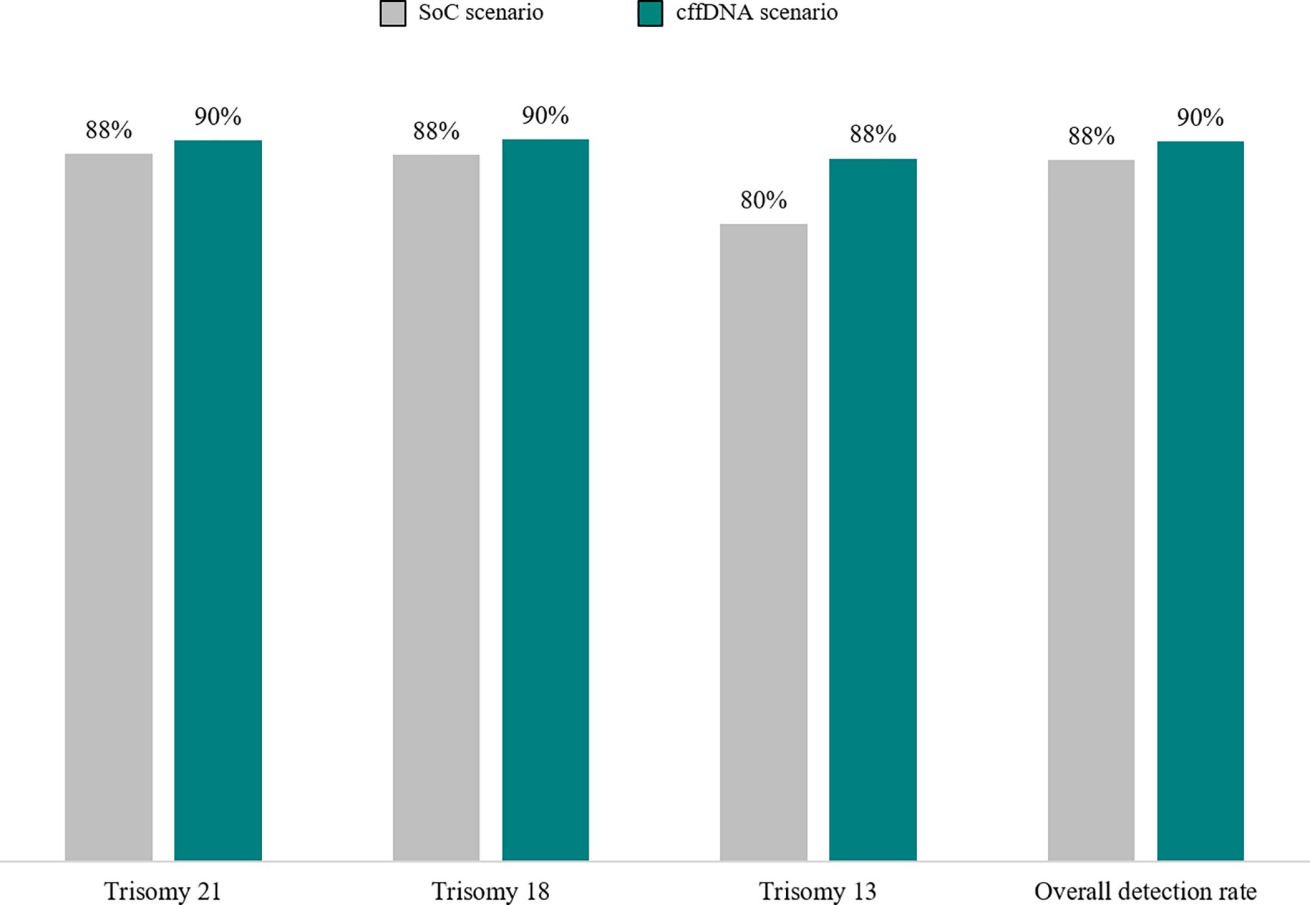
Detection rates.

According to clinical outcomes resulting from the health economics model, adopting cffDNA testing as a second line test produces a 71% reduction in the number of invasive tests performed (from 103,840 in SoC scenario to 30,368). In addition, the introduction of the test results in a lower number of false positive compared to SoC. Consequently, the number of unnecessary invasive tests due to false positives decreases by 72% (from 102,354 in SoC scenario to 28,928). Moreover, the number of procedure related-miscarriages is about three times lower than the SoC scenario (from 519 to 152), and a decrease of the other procedure-related complications was also observed (from 2,077 in SoC scenario to 607), because of the decrease of unnecessary invasive tests. Finally, the use of cffDNA increases the overall trisomy detection rate by 2,6% (87,7% vs 90%) (see Fig 5).

**Fig 6:**
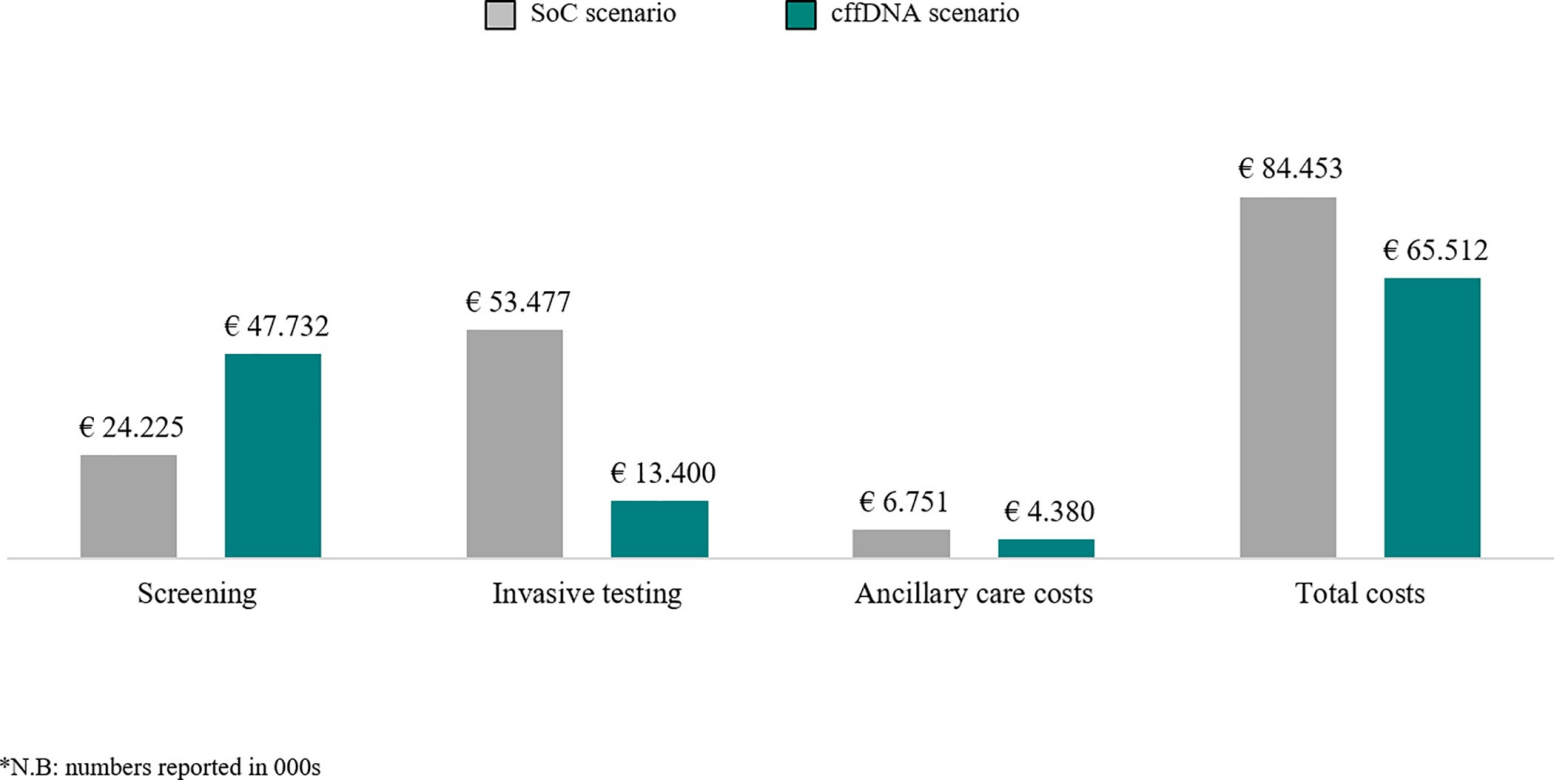
Testing costs.

In the light of the economic results regarding the adoption of the test, (i) total testing costs decrease by € 16.6 million (from € 77.7 to 61.1 million); (ii) ancillary care costs decrease by € 2.4 million (from € 6.8 to 4.4 million). Taking into account both testing costs and ancillary costs, total short term costs decrease by € 19 million (from € 84.5 to 65.5 million) (Table 4; Fig 6). Consequently, an incremental saving of € 40.49 per screened woman was estimated according to the budget impact analysis. With all these points considered, performing cffDNA in prenatal screening programs results in a lower total cost and a greater number of detected trisomies.

**Table 4:** Overall cost difference (SoC and cffDNA scenarios).

	*Costs*				*Δ Costs*			*Patients*	
	*SoC scenario*		*cffDNA scenario*					*SoC scenario*	*cffDNA scenario*
NT	€	9,303,061.76	€	10,944,778.54	€	1,641,716.78	18%	300,195	353,171
FTS	€	12,618,842.28	€	14,845,696.80	€	2,226,854.52	18%	297,194	349,640
STS	€	2,303,089.13	€	2,303,979.58	€	890.45	0%	98,718	98,756
NIPT	€	-	€	19,637,279.18	€	19,637,279.18	100%	0	67,421
Total Screening procedure	€	24,224,993.17	€	47,731,734.10	€	23,506,740.93	97%	696,107	868,988
	€								
Invasive procedure	€	53,477,070.22	€	13,399,861.63	€	-40,077,208.59	-75%	103,840	30,368
**Total Testing Costs**	**€**	**77,702,063.39**	**€**	**61,131,595.73**	**€**	**-16,570,467.65**	**-21%**		
Other procedure-related complications:									
Leakage of amniotic fluid	€	1,428,833.20	€	417,859.64	€	-1,010,973.56	-71%	1,038	304
Rare complications	€	2,546,147.54	€	744,616.16	€	-1,801,531.38	-71%	1,038	304
Miscarriages	€	550,869.20	€	161,100.68	€	-389,768.51	-71%	519	152
Terminations	€	1,497,587.50	€	1,451,338.88	€	-46,248.62	-3%	1,411	1,368
Genetic counseling	€	235,863.26	€	520,635.03	€	284,771.78	121%	11,416	25,200
Additional physician visit for high risk screens	€	491,286.65	€	1,084,446.32	€	593,159.67	121%	38,055	84,000
**Total Ancillary Care Costs**	**€**	**6,750,587.35**	**€**	**4,379,996.72**	**€**	**-2,370,590.63**	**-35%**		
**Total Short Term Costs**	**€**	**84,452,650.74**	**€**	**65,511,592.46**	**€**	**-18,941,058.28**	**-22%**		

**Fig 7:**
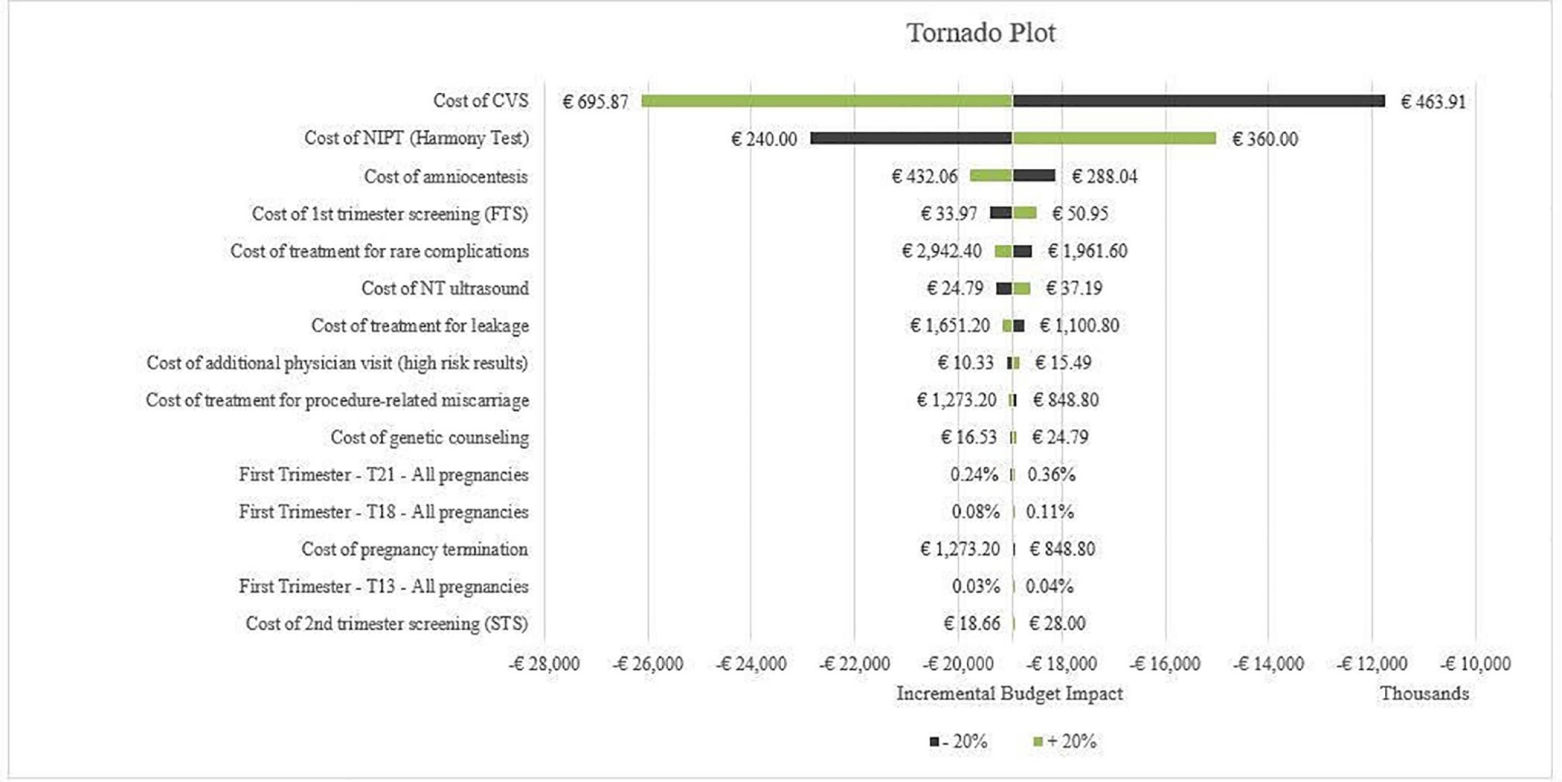
Sensitivity analysis.

Sensitivity analysis was performed to test the robustness of model results. A one-way sensitivity analysis has been performed increasing and decreasing by 20% the input variables that could significantly change the economic impact of the introduction of Harmony Prenatal test as a contingent screening for pregnant women resulted at risk from the SoC (Fig 7). The analysis suggested that 1-year net savings is sensitive to change in cost of CVS, in incidence of Trisomy 21 and in cost of Harmony Prenatal Test. After the variation of the parameters, 1-year savings range between a minimum of € 11.753.817 and a maximum of € 26.128.300, confirming the robustness of the budget impact analysis.

## 4. Discussion

According to the Italian NHS perspective, the introduction of cffDNA as a second line or contingent test compared to the current standard practice results in an increase in the detection rate of trisomies of 3%, a reduction in the number of invasive tests performed of 71%, a decrease in total short term costs of 22%.

The improvement of the detection rate concerning the screening practices may result in two principal issues: to increase screening uptake and to decrease complications and miscarriages. The strengthening of the current screening practice may result in an increase in the number of pregnant women undergoing screening procedures. As a matter of fact, pregnant women presenting for the first time may choose to undergo screening procedures instead of undergoing directly invasive tests. Moreover, increasing the detection rate causes a considerable decrease in performed invasive tests. Consequently, since invasive testing relates to a percentage of 0.5% of miscarriages [18], procedures related to miscarriages nationally decreased by 71%.

According to the analysis of the SoC scenario and the cffDNA scenario, a research was carried out to assess the impact on the budget, resulting from the possible commercialization of the Harmony Prenatal Test, under a reimbursed setting. The analysis of total short-term costs in the cffDNA scenario, with the introduction of cffDNA-based contingent testing, showed that the total short-term costs of fetal trisomy screening, before delivery, was € 65,511,592, resulting in savings when compared to the total short-term costs in the SoC scenario equal to € 84,452,651. Therefore, it was possible to conclude that the adoption of the Harmony Prenatal Test, as a second line test for women with a trisomy risk ≥ 1:1,000 after SoC screening, implies a reduction of € 18,941,058 in the expenditure charged to NHS. For both clinical and economic reasons, the adoption of Harmony Prenatal Test under a reimbursement setting would be a cost-saving choice.

Previous publications confirm that implementation of cffDNA-based contingent screening in public setting may be a cost-effective solution also in other countries with different NHS structure [23]. Additionally, a recent publication about introduction of cffDNA-based first line screening shows better clinical outcomes when this is compared to standard of care screening. At the appropriate cost, it could be also a cost-effective solution [24].

The study has a number of limitations: (1) inputs of percentage of patient flows are mainly an average value obtained according to interviews with national experts; (2) the risk cut-off 1:1,000 is the only one considered in this model; however, other risk cut-offs may be implemented according to screening procedures (e.g. 1:100, 1:300); (3) first line cffDNA testing was not considered in the model, even if it would result in better detection rates, as it would require a bigger economical investment by the NHS; (4) since the Italian NHS is based on regional level decentralization and the provided health services are not homogeneous among regions, the patient pathways may also vary across the country [25]. However, the limitations of the model are not expected to significantly impact the clinical and economic outcomes.

The main strength of the current study is the fact that it analyses short-term costs and clinical outcomes from the perspective of a public health system at a national level.

## 5. Conclusion

NHS adoption of the Harmony Prenatal Test would represent a benefit for the mother and fetus with the possibility of avoiding invasive diagnostic testing, becoming a less invasive screening choice. It would also allow a better risk stratification and would make it possible to improve pregnant women’s management, resulting in a reduction of the associated healthcare costs. Such an approach drastically reduces the use of invasive diagnosis, resulting in a decrease of the number of miscarriages associated with fetal/placental tissue sampling techniques and the consequent, though rare, complications. Therefore, in the context of prenatal screening for fetal trisomies 21, 18, 13 in the perspective of a National Health System, the possible benefits for pregnant women are twofold.
